# A potential diagnostic marker for ovarian cancer: Involvement of the histone acetyltransferase, human males absent on the first

**DOI:** 10.3892/ol.2013.1380

**Published:** 2013-06-07

**Authors:** NING LIU, RUI ZHANG, XIAOMING ZHAO, JIAMING SU, XIAOLEI BIAN, JINSONG NI, YING YUE, YONG CAI, JINGJI JIN

**Affiliations:** 1 Department of Gynecological Oncology, The First Clinical Hospital of Jilin University, Changchun, Jilin 130021, P.R. China; 2 College of Life Science, Jilin University, Changchun, Jilin 130012, P.R. China; 3 Department of Pathology, The First Clinical Hospital of Jilin University, Changchun, Jilin 130021, P.R. China

**Keywords:** histone acetyltransferase, ovarian cancer, human males absent on the first

## Abstract

Human males absent on the first (hMOF), a human ortholog of the *Drosophila* MOF protein, is responsible for histone H4 lysine 16 (H4K16) acetylation in human cells. The depletion of hMOF leads to a global reduction in histone H4K16 acetylation in human cells, genomic instability, cell cycle defects, reduced transcription of certain genes, defective DNA damage repair and early embryonic lethality. Studies have shown that abnormal hMOF gene expression is involved in a number of primary cancers. The present study examined the involvement of hMOF expression and histone H4K16 acetylation in clinically diagnosed primary ovarian cancer tissues. Clinically diagnosed frozen primary ovarian cancer tissues were used for polymerase chain reaction (PCR), quantitative PCR (qPCR), western blotting and immunohistochemical staining approaches. A PCR analysis of mRNA expression in 47 samples revealed a downregulation of hMOF mRNA in 81% of patients, whereas only 13% of patients demonstrated upregulation. qPCR was used to validate the frequent downregulation of hMOF expression in the primary ovarian cancer tissues. As expected, the analysis of hMOF expression in 57 samples revealed that hMOF mRNA expression was significantly downregulated (>2-fold decrease) in 65% of patients, while a <2-fold reduction of hMOF was observed in 10.5% of patients. Furthermore, the expression of hMOF-regulated human leukocyte antigen (HLA) complex 5, (HCP5), was also found to be downregulated in >87% of patients with a decrease in hMOF. hMOF and its regulated gene, HCP5, are frequently downregulated in human ovarian cancer, suggesting that hMOF may be involved in the pathogenesis of the disease.

## Introduction

Human males absent on the first (hMOF), also known as Moz-Ybf2/Sas3-Sas2-Tip60 1 (MYST1), is the human ortholog of the *Drosophila* MOF protein. The protein contains a chromodomain and acetyl-CoA binding motif, which is one of the key components of the male specific lethal (dMSL) complex (1). Biochemical purifications have revealed that hMOF forms at least two distinct multiprotein complexes in mammalian cells. The hMSL complex is responsible for the majority of histone H4 acetylation at lysine (K) 16 (2,3), while the hNSL (non-specific lethal) complex reveals a broad substrate specificity, which is able to acetylate histone H4 at K5, 8 and 16 (4). Although the functions of the two complexes in human cells are unclear, their ability to acetylate histone H4 at K16 indicates the significance of H4K16 acetylation in cells. It has been reported that the depletion of hMOF leads to a global reduction in histone H4K16 acetylation in human cells, in addition to genomic instability, spontaneous chromosomal aberrations, cell cycle defects, reduced transcription of certain genes, defective DNA damage repair and early embryonic lethality (4–9). This suggests a critical role for hMOF in fundamental processes, including gene transcription, cell proliferation, differentiation and the DNA repair response.

Ovarian cancer is the most common cause of cancer-related mortality from gynecological tumors (10). Due to difficulties in early detection, the majority of ovarian cancers are diagnosed at advanced stages. Studies have identified various biomarkers for diagnosing and predicting the prognosis of ovarian cancer, including the potential tumor hypoxic marker, hypoxia inducible factor-1α (HIF1α), and its regulated genes, vascular endothelial growth factor (VEGF), carbonate anhydrate IX (CA9) and stanniocalcin 1 (STC1) (11–13). However, the markers are not specific or sensitive enough to accurately predict the survival of patients with ovarian cancer (14,15). Studies have indicated that epigenetic alterations play a significant role in carcinogenesis and more recent studies have involved the use of global histone modifications as predictors of cancer recurrence in various tumor entities (16,22–24). Although the role of hMOF and its corresponding modification in transcription regulation is not completely understood, the abnormal expression of H4K16 has been identified in a number of primary cancer tissues. The expressional behavior of hMOF varies in different primary cancers. Frequent downregulation of hMOF expression has been identified in primary breast cancer and medulloblastoma (17). In contrast, hMOF was demonstrated to be overexpressed in non-small cell lung carcinoma tissues (25). In a previous study, we also showed that hMOF gene expression levels were frequently downregulated in renal cell carcinoma (RCC) (21). hMOF protein expression correlates with histone H4K16 acetylation. The previous observations strongly suggest that the histone acetyltransferase (HAT), hMOF, and the corresponding histone H4K16 acetylation may be involved in tumorigenesis. However, little is known with regard to the role of hMOF and its corresponding modification in ovarian carcinomas. The present study examined hMOF mRNA and protein expression levels in primary ovarian carcinomas using quantitative polymerase chain reaction (qPCR), western blotting and immunohistochemistry. Furthermore, the mRNA expression levels of the hMOF-regulated non-protein coding human leukocyte antigen (HLA) complex P5 (HCP5) were examined in ovarian cancer tissues.

## Materials and methods

### Antibodies and tissue collection

Anti-H4K16Ac (H9164) and anti-MYST1 (SAB4503328) polyclonal antibodies were purchased from Sigma-Aldrich (St Louis, MO, USA). Rabbit polyclonal anti-GAPDH and anti-hMOF were raised against bacterially-expressed proteins (Jilin University, Changchun, Jilin, China). Human clinical ovarian cancer and normal tissues were collected from patients with primary ovarian cancer, who underwent radical ovarian tumor surgery at The First Clinical Hospital of Jilin University (Changchun, Jilin, China) between January 2010 and July 2012. Approval for the study was obtained from the Ethics Committee of The First Clinical Hospital of Jilin University and all patients provided their informed consent. All the tissues that were removed during the surgery were frozen immediately in liquid nitrogen and stored at −80°C. Patient medical records, including tumor staging, pathological diagnosis and surgical records, were reviewed. No patients were administered chemotherapy or radiotherapy prior to surgery.

### PCR

Total RNA from ovarian cancer and normal tissues were isolated using TRIzol^®^ LS Reagent (Invitrogen, Carlsbad, CA, USA). A total of 1 *μ*g RNA from each sample was used as a template to produce cDNA using the PrimeScript 1st Strand cDNA Synthesis kit (Takara, Shiga, Japan). hMOF, CA9, VEGF, HIF1α, hSTC1 and GAPDH mRNA levels were analyzed by PCR using the C1000™ Thermal Cycler (Bio-Rad, Hercules, CA, USA) and by qPCR using Real Time PCR Detector Chromo 4 (Bio-Rad). All PCR reactions were performed under the following conditions: An initial denaturation step at 95°C for 3 min, followed by 35 cycles of denaturation at 95°C for 30 sec, annealing at 60°C for 30 sec and extension at 72°C for 30 sec. The primer sets that were used for the PCR were: GAPDH forward, 5′-ATCACTGCCACCCAGAAGAC-3′ and reverse, 5′-ATGAGGTCCACCACCCTGTT-3′, yielding a 460 bp product; hMOF forward, 5′-GGC TGGACGAGTGGGTAGACAA-3′ and reverse, 5′-TGGTGA TCGCCTCATGCTCCTT-3′, yielding a 227 bp product; CA9 for wa rd, 5′- G CAG GAG GAT TCCCCCT TG-3′ and reverse, 5′-GGAGCCTCAACAGTAGGTAGAT-3′, yielding a 185 bp product; VEGF forward, 5′-GAACTT TCTGCTGTCTTGGGTGCAT-3′ and reverse, 5′-GGTCTG CATTCACATTTGTTGTGCTG-3′, with a 392 bp product; HIF1α forward, 5′-GCACAGGCCACATTCACG-3′ and reverse, 5′-TGAAGATTCAACCGGTTTAAGGA-3′, yielding a 520 bp product; hSTC1 forward, 5′-CAC ACCCACGAGCTGACTTC-3′ and reverse, 5′-TTATGCACT CTCATGGGATGTGCG-3′, yielding a 130 bp product; and HCP5 forward, 5′-GACTCTCCTACTGGTGCTTGGT-3′ and reverse, 5′-CACTGCCTGGTGAGCCTGTT-3′, yielding a 240 bp product.

### Western blotting

Ovarian cancer or normal tissue samples (200 mg) were homogenized with liquid nitrogen and solubilized in 200 *μ*l cold PBS containing 1.0% Nonidet P-40, 0.5% Na-deoxycholate, 0.1% SDS, 0.05 mM PMSF and a protease inhibitor cocktail. The homogenate was swirled and kept on ice for 30 min. Whole cell extracts were prepared by sonication (Scientz-IID; Ningbo Scientz Biotechnology Co., Ltd., Ningbo, China) for 10 sec with 50% duty cycle and centrifugation at 13,400 x g for 30 min. The amount of total proteins in the resulting supernatant was measured according to the instructions from the Bio-Rad Protein Assay kit (500-0201). Equal total amounts of the denatured proteins were separated using 12% SDS polyacrylamide gel electrophoresis (SDS-PAGE). The specific proteins were detected by immunoblotting using hMOF, H4K16Ac and GAPDH polyclonal antibodies.

### Immunohistochemical staining

Formalin-fixed and paraffin-embedded ovarian cancer tissue blocks were obtained from The First Clinical Hospital of Jilin University. The tissue blocks were sectioned and deparaffinized in xylene and rehydrated through a graded ethanol series. The tissue slides were then subjected to antigen retrieval by boiling in 0.01 M sodium citrate buffer (pH 6) in a microwave oven for 10 min. Endogenous peroxidase activity was blocked by incubation for 10 min in 3% hydrogen peroxide in methanol. Finally, the reactions were detected using the DAB detection kit (Dako, Carpinteria, CA, USA). Anti-MYST1 (SAB4503328) and acetylated H4K16 polyclonal antibodies (H9164) were used at a 1:500 dilution. The MYST1 protein expression status and the histone H4K16 acetylation levels were estimated in a four-step scale (19).

### RNAi treatment and DNA microarray

HeLa cells were cultured in 6-well tissue culture plates (∼2×10^5^ cells/well) in DMEM medium (Sigma) containing 5% glucose and 10% fetal bovine serum. The cells were transfected with 20 nM hMOF siRNA (D-014800, Dharmacon, Lafayette, CO, USA) and non-targeting siRNA (D-001206, Dharmacon). At 24 h post-transfection, the cells were split into new 6-well plates for immunoblotting, PCR and DNA microarray analysis. Subsequent to 24 h, the cells were harvested and lysed. Whole cell extracts were prepared from two wells of a 6 well plate by adding 4 × SDS sample buffer, and total RNA was isolated from one well of a 6 well plate using TRIzol^®^ LS Reagent (Invitrogen). In addition, cells from one well of the 6-well plate were rinsed twice with warm PBS and harvested. The cells were then stored in an RNA hold solution (ER501-01; Beijing Transgen Biotech Co., Ltd., Beijing, China) and sent to OneArray by Phalanx Biotech Group (Belmont, CA, USA) for DNA microarray analysis.

### Chromatin immunoprecipitation (ChIP)

One or two 10-cm dishes containing ∼1×10^7^ HeLa cells grown to ∼80% confluence were used for each ChIP. The cells were cross-linked with 5 ml 1% formaldehyde in PBS for 15 min at room temperature, followed by incubation with 125 mM glycine for 5 min. In order to shear the DNA to lengths of 200–1,000 base pairs, the cell lysates were sonicated using a Scientz-IID for 5×60 sec, with a one sec interval between each round, at a setting of 45% duty, level 2. Equal amounts of sonicated chromatin from each sample were incubated at 4°C overnight with 5–10 *μ*g of antibodies against H4K16Ac (H9164, Sigma) or hMOF rabbit polyclonal antibodies. Total rabbit IgG (sc-2027, Santa Cruz Biotechnology, Inc., Santa Cruz, CA, USA) and pre-immune serum were used as controls. Following 24 h of incubation, 50 *μ*l protein A agarose containing salmon sperm DNA (10 *μ*g) and BSA (25 *μ*g; 50% Slurry) were added to the mixture and further incubated for 2.5 h at 4°C to collect the agarose/antibody/protein complex. The protein A agarose/antibody/protein complex was washed for 5 min on a rotating platform with 1 ml buffer according to the order of Low Salt- (0.1% SDS, 1% Triton-100, 2 mM EDTA, 20 mM Tris, pH 8.0 and 150 mM NaCl) High Salt- (0.1% SDS, 1% Triton-100, 2 mM EDTA, 20 mM Tris, pH 8.0 and 500 mM NaCl) LiCl- (0.25 M LiCl, 1% NP-40, 1% NaDOC, 1 mM EDTA and 10 mM Tris, pH 8.0) TE- (10 mM Tris, pH 8.0 and 1 mM EDTA) TE (10 mM Tris, pH 8.0 and 1 mM EDTA). Finally, the washed beads were eluted using a 480 *μ*l elution buffer containing 0.1 M NaHCO_3_ and 1% SDS. DNA was extracted by phenol/chloroform and precipitated using ethanol. Amplification of 1–2 *μ*l immunoprecipitated or 100-fold diluted input DNA was performed using Real Time PCR Detector Chromo 4 (Bio-Rad). Each experiment was performed independently, 2–3 times. All ChIP signals were normalized to the total input. The primer sets of the HCP5 promoter (−142 to +50) were forward, 5′-TCCACCTTTCCCAACCTGTGTC-3′ and reverse, 5′-GGACTCCATGACCCGCAACC-3′.

### Statistical analysis

The gene expression signals on the 2% agarose gel and western blot images were scanned and quantified with Quantity One Basic software (Bio-Rad). The differences in the expression of genes and proteins between ovarian cancer and normal tissues were statistically analyzed. Statistical analysis was completed with SPSS 17.0 (SPSS, Inc., Chicago IL, USA). Statistical comparisons were analyzed using the student’s t-test. P<0.05 was considered to indicate a statistically significant difference.

## Results

### A reduction in hMOF mRNA expression levels is observed in ovarian cancer tissues

**Figure 1.**
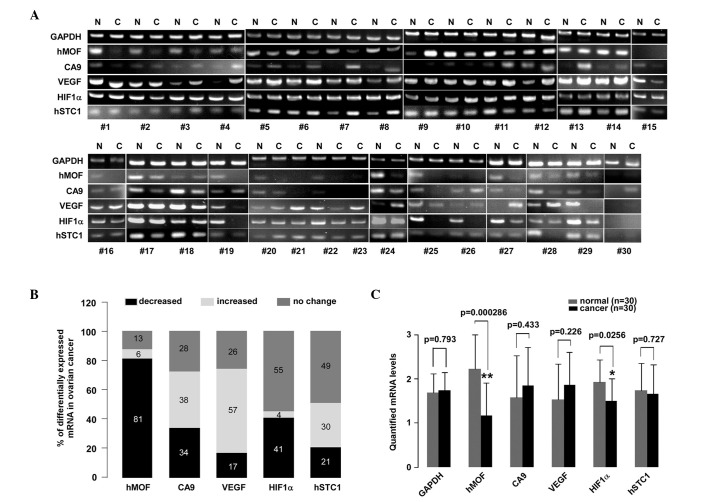
A reduction in hMOF mRNA levels is observed in human ovarian cancer. (A) PCR analysis of 47 clinical ovarian cancer tissues. Total RNA was isolated from the tissues using TRIzol. The PCR assay was performed to detect the mRNA expression levels of hMOF, CA9, VEGF, HIF1α and hSTC1 in clinical ovarian cancer and normal ovarian tissues. The PCR products were then separated by electrophoresis on a 2% agarose gel. The DNA fragments were visualized and photographed under ultraviolet light with ethidium bromide. The mRNA levels from 37 ovarian cancer tissues were compared with corresponding contralateral ovarian normal tissues. However, 10 clinical ovarian cancer tissues were missing contralateral ovarian normal tissues and were compared with non-corresponding normal ovarian tissues. (B) Summarization of the PCR results. The 100% stacked column charts were used to compare the case numbers of differentially-expressed mRNAs in the ovarian cancer tissues. The total case numbers of differentially-expressed mRNAs (increased, decreased and no change) in the ovarian cancer tissues is equal to 100%. (C) Statistical analysis of quantified mRNA levels between the ovarian cancer and normal tissues. The mRNA expression signals shown in (A) were quantified by densitometry using Quantity One Basic Software. The significant difference is expressed as ^*^P<0.05, ^**^P<0.01. hMOF, human males absent on the first; PCR, polymerase chain reaction; CA9, carbonate anhydrate IX; VEGF, vascular endothelial growth factor; HIF1α, hypoxia-inducible factor-1α; hSTC1, human stanniocalcin 1; N, normal tissue; C, cancer tissue.

In order to identify the expression levels of several cancer-related genes in the pathogenesis of primary ovarian cancer, the present study examined the mRNA levels of hMOF, CA9, VEGF, HIF1α and hSTC1 in 47 patients with pathologically-diagnosed ovarian cancer using PCR. Fig. 1A displays a section of the PCR results (n=30). Compared with the contralateral normal ovarian tissues or the normal ovarian tissues from an alternative patient, the mRNA expression levels of hMOF, CA9, VEGF, HIF1α and hSTC1 in the ovarian cancer tissues revealed varying behaviors on the DNA agarose gel. Among them, the hMOF gene expression levels showed a decreasing tendency in the ovarian cancer tissues. The quantified mRNA levels are shown in Fig. 1C. The gene expression levels of hMOF and HIF1α were markedly decreased in the ovarian cancer tissues compared with the normal tissues (P<0.01 and P<0.05, respectively). However, no significant differences were observed in VEGF, CA9 and hSTC1 expression. An analysis of mRNA expression in 47 samples revealed the downregulation of hMOF mRNA in 81% (38/47) of patients, whereas only 13% (6/47) showed upregulation and 6% (3/47) showed no change (Fig. 1B). In contrast with hMOF, the expression levels of VEGF were upregulated in more than half of the cases (27/47, 57%), while only 17% (8/47) of patients exhibited downregulation and 26% (12/47) exhibited no change. Although HIF1α mRNA expression was downregulated in 40% (19/47) of patients, the majority of the cases did not change (26/47, 55%). Almost half of the patients (23/47, 49%) revealed no changes in the mRNA expression levels of hSTC1. The percentage change between the upregulation and downregulation was 30% (14/47) and 21% (10/47), respectively. There were no significant changes in the number of cases of varying CA9 expression. The percentage changes in the upregulation, downregulation or no change in CA9 mRNA expression were 38%, 34% and 28%, respectively.

### Frequent downregulation of hMOF in ovarian cancer tissues is confirmed using qPCR

**Figure 2.**
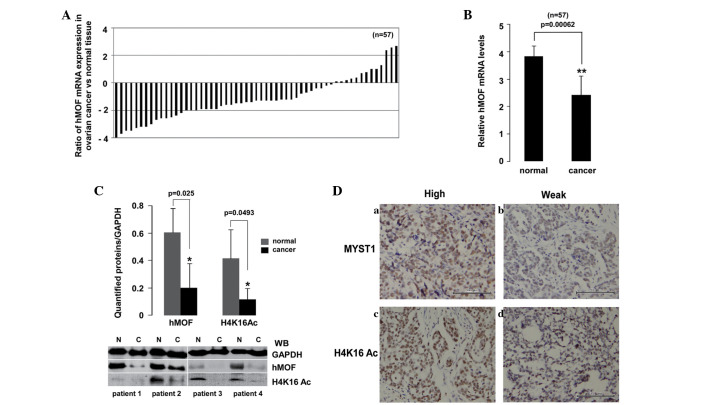
Downregulation of hMOF mRNA expression in ovarian cancer tissues confirmed using qPCR and immunoblotting. (A) Expression patterns of hMOF in clinical ovarian cancer tissues. Total RNA was isolated from 57 clinical ovarian cancer and 15 normal tissues (8/15 cases were the contralateral normal tissues). The relative mRNA expression levels of hMOF were analyzed using qPCR. Expression is displayed as a ratio of hMOF gene expression in the ovarian cancer versus normal tissues. Each bar is the log2 value of the ratio of hMOF expression levels between the ovarian cancer and normal tissues. Bar value >1 represents >2-fold increase, whereas bar value <1, represents >2-fold decrease. (B) Statistical analysis of the qPCR data. Each bar represents the mean of three independent experiments. The significant difference is expressed as ^**^P<0.01. (C) Four randomly selected, pathologically diagnosed ovarian cancer and contralateral normal tissues from the same patients were used. A whole cell extract was prepared from the tissues and equivalent total protein amounts were subjected to SDS-PAGE in 12% gels. Proteins were detected by western blotting with anti-hMOF, H4K16Ac and GAPDH antibodies (lower panel). Western blotting images were quantified using Quantity One software and normalized by GAPDH levels. The significant difference is expressed as ^*^P<0.05. (D) Immunohistochemical staining for (a and b) hMOF and (c and d) H4K16Ac in ovarian cancer tissues (×200). (a and c) High or (b and d) low hMYST1 and H4K16Ac expression levels in ovarian cancer tissues. hMOF, human males absent on the first; qPCR, quantitative polymerase chain reaction; H4K16, histone H4 lysine 16; MYST1, Moz-Ybf2/Sas3-Sas2-Tip60 1; N, normal tissue; C, cancer tissue.

The results of the PCR analysis clearly revealed a downregulation of hMOF gene expression in ovarian cancer. To further validate the frequent downregulation of hMOF mRNA expression in primary ovarian cancer, an additional 57 clinically diagnosed ovarian cancer tissues and 15 normal tissues were used in this experiment (8/15 cases were contralateral normal ovarian tissues). The expression levels of hMOF were measured using qPCR. As shown in Fig. 2A, the analysis of mRNA expression in the 57 samples revealed a significant (>2-fold decreased) downregulation of hMOF mRNA in 65% (37/57) of patients, whereas 11% (6/57) of patients showed significant (>2-fold increased) upregulation of hMOF. In addition, a <2-fold reduction of hMOF was observed in 11% (6/57) of patients. In contrast, 12% (7/57) of patients showed a <2-fold elevation of hMOF. The significant differences in hMOF expression between ovarian cancer and normal tissues were analyzed using Student’s t-test. As shown in Fig. 2B, the hMOF expression levels were significantly reduced in ovarian cancer (P<0.01). In order to determine whether the reduction of hMOF expression resulted in decreased hMOF protein levels, four randomly selected ovarian cancer tissues and matched contralateral normal ovarian tissues were used (Fig. 2B). As shown in the lower panel, aliquots of whole cell extract from the tissues were analyzed by western blotting using the indicated antibodies. As expected, the level of hMOF protein expression was decreased in the ovarian cancer tissues compared with the matched normal tissues. Simultaneously, the acetylation status of histone H4K16 was also significantly reduced or lost in the ovarian cancer samples. To further confirm this result, immunohistochemical staining was performed for hMOF and histone H4K16 acetylation in the formalin-fixed, paraffin-embedded tissue sections from 20 patients. However, the immunohistochemical staining of certain cases failed. Therefore, no immunohistochemistry analysis data are presented. As an example, high or low immunohistochemical staining for hMOF and H4K16Ac in ovarian cancer is shown in Fig. 2D.

### HCP5, an hMOF-regulated gene, is also frequently downregulated in ovarian cancer tissues

**Figure 3.**
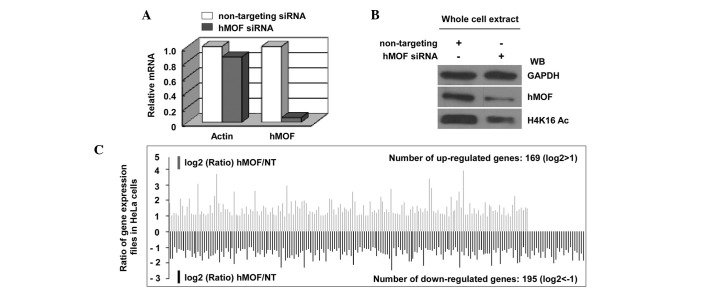
Gene expression profiles in hMOF siRNA knockdown HeLa cells. (A) Relative mRNA levels markedly decreased following the specific knockdown of hMOF. The HeLa cells were transfected with hMOF siRNAs or a non-targeting (NT) control siRNA. At 48 h post-transfection, hMOF mRNA levels were measured by qPCR. Actin mRNA was used to assess the integrity of the RNA. (B) hMOF protein expression and its corresponding modifications were down-regulated by transfecting HeLa cells with hMOF siRNAs. Equivalent total protein amounts of whole cell extracts from the cells treated with hMOF siRNAs or NT siRNAs were subjected to SDS-PAGE in 12% gels, and the proteins were detected by western blotting using the indicated antibodies. (C) Gene expression profiles in hMOF siRNA knockdown HeLa cells. The gray bars represent the upregulated genes (log2>1), while the black bars represent the downregulated genes (log2<−1). Each bar is the log2 value of the ratio of gene expression in hMOF siRNA knockdown HeLa cells. hMOF, human males absent on the first; qPCR, quantitative polymerase chain reaction; H4K16, histone H4 lysine 16; WB, western blotting.

**Figure 4.**
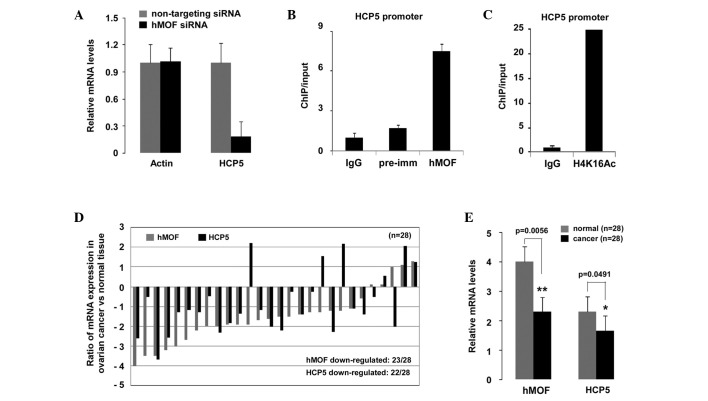
HPC5, an hMOF target gene, is frequently downregulated in ovarian cancer tissues. (A) A reduction in HCP5 mRNA expression levels in hMOF siRNA knockdown cells. HeLa cells were transfected with hMOF or NT siRNAs. Following 48 h of transfection, the mRNA levels of HCP5 and actin were measured by qPCR. Error bars represent the standard error of the mean of three independent experiments. (B and C) hMOF colocalizes with H4K16Ac at HCP5 promoter. ChIP assays using transfected hMOF or NT siRNA HeLa cells were analyzed by qPCR. Bar graphs show the ratio of ChIP signals that were normalized to the input DNA. (D) HCP5 mRNA expression patterns in ovarian cancer tissues. 28 randomly selected clinical ovarian cancer and contralateral normal tissues were used. The HCP5 and hMOF mRNA expression levels in ovarian cancer were analyzed by qPCR. The y-axis indicates the log2 value of the ratio of HCP5 and hMOF expression levels between the cancer and normal tissues from the same patients. (E) Statistical analysis of qPCR results. Each bar represents the mean of three independent experiments. The significant difference is expressed as ^*^P<0.05 and ^**^P<0.01. hMOF, human males absent on the first; HCP5, human leukocyte antigen (HLA) complex P5; NT, non-targeting; qPCR, quantitative polymerase chain reaction; ChIP, chromatin immunoprecipitation.

In order to confirm that hMOF was downregulated in ovarian cancer, hMOF target genes were screened from gene expression profiles in hMOF siRNA knockdown HeLa cells. The effect of siRNA interference was confirmed using qPCR and immunoblotting (Fig. 3A and B). As a result of the DNA microarray analysis by the Phalanx Human OneArray™ (HOA 5.2: Phalanx Biotech Group, Inc.), a total of 364 genes were shown to be differentially-expressed between the hMOF siRNA knockdown and non-targeting (NT) siRNA-treated HeLa cells, of which 169 genes were upregulated and 195 genes were downregulated by >2-fold (Fig. 3C; Table I). HCP5 was confirmed as one of the hMOF downregulated genes by qPCR and ChIP. As shown in Fig. 4A, the relative mRNA levels of HCP5 in the hMOF siRNA knockdown HeLa cells were markedly decreased compared with those of the NT siRNA-treated cells. To determine whether hMOF was recruited to the HCP5 promoter, ChIP experiments were performed using chromatin from NT siRNA or hMOF siRNA knockdown HeLa cells. As expected, hMOF-specific and histone H4K16Ac antibodies precipitated a DNA fragment encompassing the HCP5 promoter (Fig. 4B and C), suggesting that HCP5 was an hMOF target gene. To further investigate whether the mRNA expression levels of HCP5 were also affected in hMOF-downregulated human ovarian cancer tissues, 28 clinically diagnosed ovarian cancer tissues were selected. The total mRNA expression levels of hMOF and HCP5 were observed to be significantly decreased compared with those of the normal tissues (P<0.01 and P<0.05, respectively; Fig. 4E). The results are shown in Fig. 4D, where 23 of the 28 patients showed a downregulation of hMOF, among which, 20 (87%) also exhibited a reduction in HCP5 expression.

## Discussion

Histone acetylation, as one of the best-characterized epigenetic modifications, is controlled by HATs and histone deacetylases (HDACs). The balance between histone acetylation and deacetylation serves as a key epigenetic mechanism for gene expression, DNA repair, developmental processes and tumorigenesis (5–7). Thus, an imbalance may lead to abnormal cell functions and cancer. MYST1, also known as hMOF, a member of the MYST family of HATs and an epigenetic marker of active genes, is responsible for histone H4K16 acetylation in human cells (2,3). Studies have demonstrated that hMOF participates in a number of biological processes, including gene transcription, cell proliferation, differentiation and the DNA repair response (4–9). The fact that hMOF is involved in these critical cellular functions suggests that hMOF may play a significant role in tumorigenesis. Although little is known about the mechanism of hMOF in tumor development and progression, hMOF expression in clinical cancer tissues has been reported by several studies. A frequent downregulation of hMOF in primary breast carcinomas, renal cell carcinoma and medulloblastomas has been identified and the reduction in hMOF protein expression has been shown to correlate with H4K16 acetylation in those tumors (17,21). In addition, the analysis of tissue microarray slides has revealed low or absent histone H4K16 acetylation in the majority of breast cancer tissues (16). However, the expression of hMOF in non-small cell lung carcinoma tissues has been shown to be frequently elevated (25). The present study investigated the expression of the HAT, hMOF, and its corresponding H4K16 acetylation in a series of primary ovarian cancer tissues by qPCR, western blotting and immunohistochemistry. The results revealed that hMOF mRNA or protein expression was frequently downregulated in human ovarian cancer (>75%), and that hMOF protein expression was correlated with histone H4K16 acetylation in parallel. Furthermore, the hMOF-regulated gene, HCP5, was also found to be downregulated in ovarian cancer tissues. Therefore, hMOF may have a significant role in primary ovarian carcinoma tumorigenesis.

HLA is the human version of the major histocompatibility complex (MHC). The genes in this complex are categorized into classes I, II and III. MHC or HLA modulates the efficacy of cytotoxic immune responses through the presentation of tumor antigens (19). The modulation of tumor antigen-specific immune responses by an abnormal expression of HLA-class I and II molecules has been identified in a variety of carcinomas, including ovarian cancer (20). HCP5 is localized within the MHC class I region. Although the function of HCP5 is not well known, the sequence of the gene is associated with the human endogenous retroviruses, HERV-L and HERV-16 (18). In the present study, the expression of hMOF was frequently downregulated in the clinical ovarian cancer tissues (Figs. 1 and 2) and, more notably, HCP5 was presented in gene expression profiles as a hMOF-downregulated gene. Together, this suggests that HCP5, as a hMOF-target gene, may also be downregulated in ovarian cancer. This prediction was confirmed using qPCR. HCP5 gene expression was downregulated in >87% of patients with a decreased hMOF level. Although the functional mechanism between hMOF and HCP5 is unclear, a reduction in hMOF may lead to defective expression of its target genes.

In summary, frequent downregulation of hMOF and a loss of H4K16 acetylation are observed in ovarian cancer tissues. Although a large series of clinical cases and analyses of overall survival are required for further investigations, the molecular mechanism that links a loss of hMOF expression to ovarian cancer will be a promising area for further research. Combined with the results from previous studies, the present study concluded that the abnormal expression of hMOF in tumors may be a common feature, suggesting that hMOF may be a novel epigenetic biomarker for tumor diagnosis.

## Figures and Tables

**Table I.** Downregulated gene expression profiles in hMOF siRNA knockdown HeLa cells.

Gene symbol	Gene ID No.	Gene description	Fold-change
ANKRD33B	NM_001164440	Ankyrin repeat domain 33B	−3.11
PSMB 10	NM_002801	Proteasome subunit, β type, 10	−3.18
ANKRD2	NM_020349	Ankyrin repeat domain 2	−3.48
CARS2	NM_024537	Cysteinyl-tRNA synthetase 2	−3.09
RHEBL1	NM_144593	Ras homolog enriched in brain like 1	−4.61
STK36	NM_015690	Serine/threonine kinase 36	−3.64
SEMA7A	NM_003612	Semaphorin 7A, GPI membrane anchor	−3.35
CXCL10	NM_001565	Chemokine (C-X-C motif) ligand 10	−3.84
ACSL5	NM_016234	Acyl-CoA synthetase long-chain family member 5	−3.04
SAA1	NM_000331	Serum amyloid A1	−3.65
HCP5	NR_040662	HLA complex P5 (non-protein coding)	−3.19
DHX58	NR_024119	DEXH (asp-Glu-X-His) box polypeptide 58	−3.21
IFI27	NM_005532	Interferon, α-inducible protein 27	−3.85

hMOF, human males absent on the first; HLA, human leukocyte antigen.

## Abbreviations:

HAT: histone acetyltransferase;
MYST: Moz-Ybf2/Sas3-Sas2-Tip60;
HLA: human leukocyte antigen;
HCP5: HLA complex P5;
RT-PCR: reverse transcription-polymerase chain reaction;
qPCR: quantitative PCR.
