# [Corrigendum] A potential diagnostic marker for ovarian cancer: Involvement of the histone acetyltransferase, human males absent on the first 

**DOI:** 10.3892/ol.2026.15526

**Published:** 2026-03-12

**Authors:** Ning Liu, Rui Zhang, Xiaoming Zhao, Jiaming Su, Xiaolei Bian, Jinsong Ni, Ying Yue, Yong Cai, Jingji Jin

Oncol Rep 6: 393–400, 2013; DOI: 10.3892/ol.2013.1380

Following the publication of the above paper, an interested reader drew to the authors’ attention that, regarding the PCR analysis of clinical ovarian cancer tissues shown in Fig. 1A on p. 396, the GAPDH data [for the normal (N) and cancer (C) tissues] for RT-PCR samples #15 and #24 were strikingly similar, suggesting that an error had been made during the assembly of this figure.

Upon assessing their data, the authors realized that the correct GAPDH data for RT-PCR sample #15 had inadvertently been reinserted into the figure for RT-PCR sample #24. The revised version of Fig. 1, now showing the correct GAPDH data for RT-PCR sample #24 in Fig. 1A, is shown on the next page. The authors regret that this error went unnoticed before this article was published, although note that it did not grossly affect either the results or the conclusions reported in this article. All the authors agree with the publication of this Corrigendum, and thank the Editor of *Oncology Letters* for granting them the opportunity to publish this; furthermore, they apologize to the readership for any inconvenience caused.

## Figures and Tables

**Figure 1.**
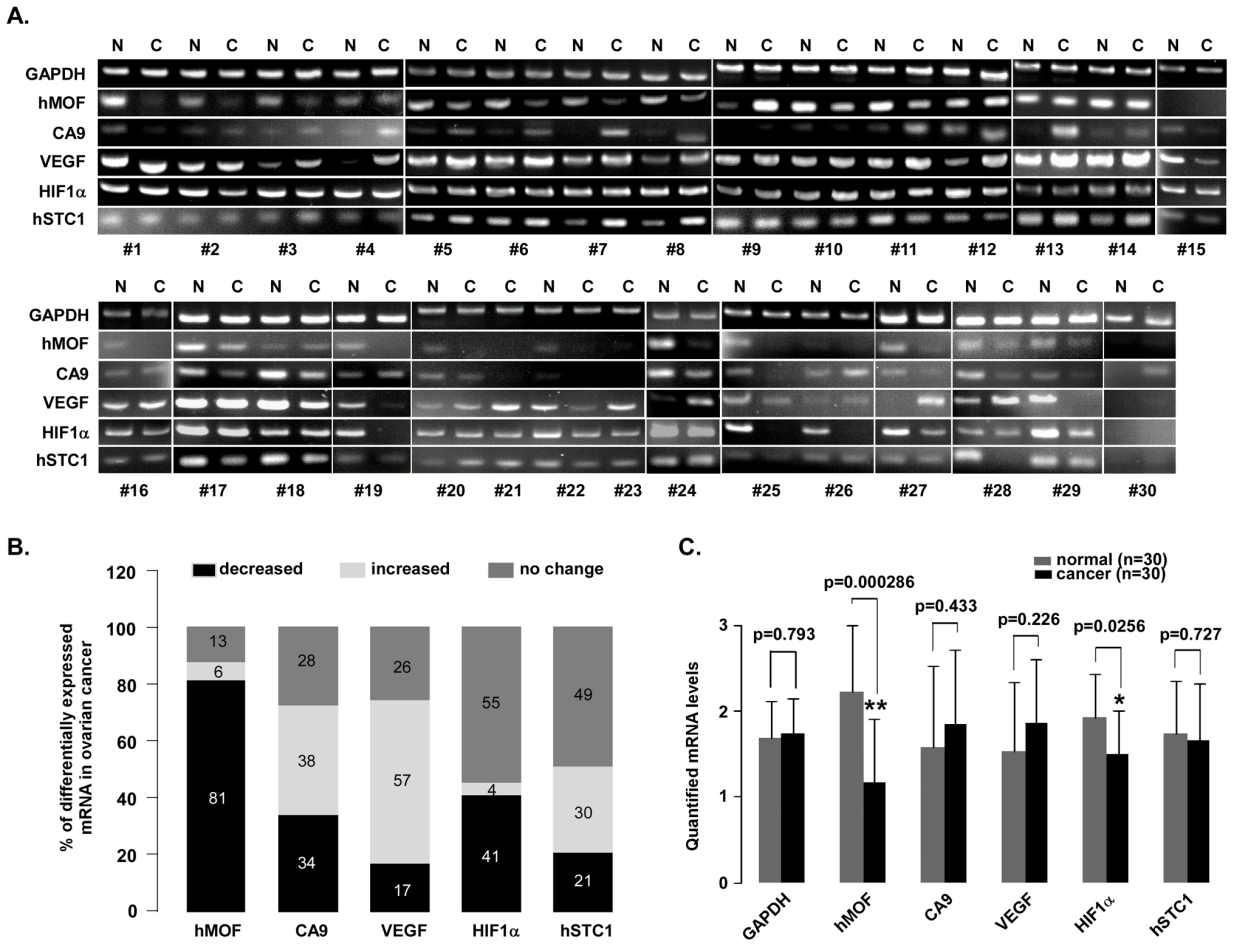
A reduction in hMOF mRNA levels is observed in human ovarian cancer. (A) PCR analysis of 47 clinical ovarian cancer tissues. Total RNA was isolated from the tissues using TRIzol. The PCR assay was performed to detect the mRNA expression levels of hMOF, CA9, VEGF, HIF1α and hSTC1 in clinical ovarian cancer and normal ovarian tissues. The PCR products were then separated by electrophoresis on a 2% agarose gel. The DNA fragments were visualized and photographed under ultraviolet light with ethidium bromide. The mRNA levels from 37 ovarian cancer tissues were compared with corresponding contralateral ovarian normal tissues. However, 10 clinical ovarian cancer tissues were missing contralateral ovarian normal tissues and were compared with non-corresponding normal ovarian tissues. (B) Summarization of the PCR results. The 100% stacked column charts were used to compare the case numbers of differentially-expressed mRNAs in the ovarian cancer tissues. The total case numbers of differentially-expressed mRNAs (increased, decreased and no change) in the ovarian cancer tissues is equal to 100%. (C) Statistical analysis of quantified mRNA levels between the ovarian cancer and normal tissues. The mRNA expression signals shown in (A) were quantified by densitometry using Quantity One Basic Software. The significant difference is expressed as *P<0.05, **P<0.01. hMOF, human males absent on the first; PCR, polymerase chain reaction; CA9, carbonate anhydrate IX; VEGF, vascular endothelial growth factor; HIF1α, hypoxia-inducible factor-1α; hSTC1, human stanniocalcin 1; N, normal tissue; C, cancer tissue.

